# The Efficacy of Edaravone (Radicut), a Free Radical Scavenger, for Cardiovascular Disease

**DOI:** 10.3390/ijms140713909

**Published:** 2013-07-04

**Authors:** Kiyoshi Kikuchi, Salunya Tancharoen, Nobuyuki Takeshige, Munetake Yoshitomi, Motohiro Morioka, Yoshinaka Murai, Eiichiro Tanaka

**Affiliations:** 1 Department of Pharmacology, Faculty of Dentistry, Mahidol University, 6 Yothe Road, Rajthevee, Bangkok 10400, Thailand; E-Mails: kikuchi_kiyoshi@kurume-u.ac.jp (K.K.); salunya.tan@mahidol.ac.th (S.T.); 2 Division of Brain Science, Department of Physiology, Kurume University School of Medicine, 67 Asahi-machi, Kurume 830-0011, Japan; E-Mail: ymurai@med.kurume-u.ac.jp; 3 Department of Neurosurgery, Kurume University School of Medicine, 67 Asahi-machi, Kurume 830-0011, Japan; E-Mails: takeshige_nobuyuki@med.kurume-u.ac.jp (N.T.); munetake06@gmail.com (M.Y.); mmorioka@med.kurume-u.ac.jp (M.M.)

**Keywords:** edaravone, cardiovascular disease, free radical scavenger

## Abstract

Edaravone was originally developed as a potent free radical scavenger, and has been widely used to treat acute ischemic stroke in Japan since 2001. Free radicals play an important role in the pathogenesis of a variety of diseases, such as cardiovascular diseases and stroke. Therefore, free radicals may be targets for therapeutic intervention in these diseases. Edaravone shows protective effects on ischemic insults and inflammation in the heart, vessel, and brain in experimental studies. As well as scavenging free radicals, edaravone has anti-apoptotic, anti-necrotic, and anti-cytokine effects in cardiovascular diseases and stroke. Edaravone has preventive effects on myocardial injury following ischemia and reperfusion in patients with acute myocardial infarction. Edaravone may represent a new therapeutic intervention for endothelial dysfunction in the setting of atherosclerosis, heart failure, diabetes, or hypertension, because these diseases result from oxidative stress and/or cytokine-induced apoptosis. This review evaluates the potential of edaravone for treatment of cardiovascular disease, and covers clinical and experimental studies conducted between 1984 and 2013. We propose that edaravone, which scavenges free radicals, may offer a novel option for treatment of cardiovascular diseases. However, additional clinical studies are necessary to verify the efficacy of edaravone.

## 1. Introduction

Cardiovascular disease is the leading cause of death worldwide in the Global Burden of Diseases, Injuries, and Risk Factors Study 2010 (GBD 2010) [1]. The World Health Organization (WHO) has estimated that 17.3 million people died from cardiovascular disease in 2008 and 23.3 million people would die from cardiovascular disease by 2030 [2]. Therefore, the treatment of cardiovascular disease needs to be improved.

A previous review estimated that more than 1000 neurovascular protective agents have been tested in animal models of acute ischemic stroke (AIS) [3]. However, none of the 114 agents that entered clinical trials were shown to be useful [4]. Several free radical scavengers, such as NXY-059, ebselen, nicaraven, and tirilazad, were assessed for their efficacy in the treatment of AIS, yet these failed to prove useful in clinical trials [5–9]. A recent systematic review and meta-analysis of 50 randomized controlled trials with 294,478 participants found no evidence to support the use of vitamins and antioxidant supplements for prevention of cardiovascular diseases [10].

Edaravone (3-methyl-1-phenyl-2-pyrazolin-5-one, MCI-186, Radicut), a strong novel free radical scavenger, was developed by Mitsubishi Tanabe Pharma Corporation (Osaka, Japan). Edaravone has been widely used in patients with AIS since April 2001 in Japan, because many clinical and experimental studies have demonstrated neurovascular protective effects [11–15]. Furthermore, edaravone has anti-apoptotic, anti-necrotic, and anti-inflammatory cytokine effects, as well as scavenging free radicals in cardiovascular diseases and stroke, showing protective effects in the heart, vessel, and brain in experimental studies [16–19]. Moreover, edaravone has preventive effects on myocardial injury following ischemia and reperfusion (I/R) in patients with acute myocardial infarction (AMI) [20].

## 2. Mechanisms of the Action of Edaravone

Edaravone has been reported to exert antioxidant effects because it can quench hydroxyl radicals and hydroxyl radical-dependent lipid peroxidation [21,22]. Edaravone reduces elevated levels of hydroxyl radicals and superoxide radicals in several models of ischemia [23,24]. In early studies of antioxidant activity of edaravone, its pKa was found to be 7.0, and the rate of oxidation for edaravone was positively correlated with pH [25,26]. The putative mechanism underlying the antioxidant action of edaravone is electron transfer from an edaravone anion to peroxyl radical, and this reaction breaks the chain oxidation of lipids. Edaravone (50 μM) was found to inhibit the aerobic oxidation of unilamellar soybean phosphatidylcholine liposomal membranes, which was initiated with either a water-soluble or a lipid-soluble initiator [26]. A previous study described the ability of edaravone to inhibit copper- and human umbilical vein endothelial cell (HUVEC)-mediated oxidation of low-density lipoprotein (LDL) *in vitro* [27]. The mechanisms of the effect of edaravone were mediated by enhancement of endothelial nitric oxide synthase (eNOS) expression in HUVECs via stabilizing eNOS mRNA and inhibition of the reduction in eNOS expression induced by oxidized LDL. The authors of the study speculated that edaravone might improve vascular blood flow via upregulation of eNOS and a decrease in LDL oxidation, which may in turn result in a protective effect in ischemic tissues. Furthermore, Tosaka *et al.* reported that edaravone might have vasoprotective effects against hydroxyl radical, and could become a valuable drug for the treatment of cerebral vasospasm in the canine basilar artery [28]. These results suggest that edaravone may reduce membrane lipid peroxidation and has a protective effect in cardiovascular disease and stroke.

Reactive oxygen species (ROS) and Ca^2+^ overload during I/R-induced cellular damage by opening the mitochondrial permeability transition pore, which is a non-specific pore in the inner mitochondrial membrane in the rat brain [29]. Kawai *et al.* reported that ROS contribute to brain injury after permanent focal ischemia and that the treatment with edaravone would be beneficial in the rat middle cerebral artery occlusion (MCAO) model [30]. Notably, edaravone attenuates the Ca^2+^-induced swelling of mitochondria in the rat brain. In addition, edaravone protects ischemic neurons from apoptosis by suppressing the expression of Fas-associated death domain protein, death-associated protein, and caspase-8 immunoreactivity in a rat MCAO model, which is a focal ischemic animal model [31]. Edaravone also has another anti-apoptotic effect, which is mediated by a decrease in the level of B-cell lymphoma 2-associated X protein immunoreactivity and by an increase in the level of B-cell lymphoma 2 immunoreactivity—an apoptosis regulator—in the rat MCAO model [32]. The protective effect of edaravone in hypoxic/ischemic injury has also been attributed to inhibition of the response to endoplasmic reticulum (ER) stress and subsequent apoptotic signaling in a focal ischemic model [33,34]. In a mouse focal ischemic model, the neuroprotective effects of edaravone were mediated via its antioxidant actions, including suppression of lipid peroxidation and oxidant-induced DNA damage [35]. In a gerbil forebrain ischemic model, edaravone also suppressed the activity of inducible nitric oxide synthase (iNOS), thereby exerting anti-inflammatory effects by inhibition of peroxynitrite production as microglial activity. Using a standard ischemia model in gerbils, Jin and colleagues showed that edaravone reduced edema and increased cerebral blood flow following ischemia [36]. Data from several animal models suggest that edaravone suppresses cerebral edema in hypoxic/ischemic conditions [14,37]. This effect is attributed to edaravone-mediated inhibition of vascular endothelial growth factor expression and aquaporin-4 expression in astrocytes. In addition to the inhibition of ROS generation, edaravone reduces the amount of ROS-induced inflammatory reactions in cerebral ischemia [38]. Oxidative stress activates nuclear factor-κB (NF-κB) and several mediators of inflammation (e.g., iNOS, cytokines, and cyclooxygenase-2) that are known to cause delayed damage to the ischemic area in stroke patients and models of stroke [39–46]. In cases of ischemic injury, edaravone can also reduce iNOS expression and suppress neutrophil activation and the accumulation of lipid peroxidation products and 4-hydroxy-2-nonenal (HNE)-modified proteins in mice [35,47]. Taken together, these results suggest that edaravone may have protective effects against apoptosis and inflammation, which are induced by Ca^2+^ overload, ROS generation, and iNOS expression, in cerebral ischemia.

Recent discoveries indicate many new benefits of edaravone in cerebral ischemia. Several lines of evidence show that edaravone induces neurovascular protection after cerebral ischemia. This is associated with the release of high mobility group box 1 (HMGB1) protein from neuronal cells or myocardium after ischemic insults [12,48]. An elevation in HMGB1 levels is associated with poor clinical outcomes [49,50]. Edaravone rescues rats from neuronal death after cerebral ischemia by attenuation of the release of HMGB1 from neuronal cells in the rat MCAO model [12]. Edaravone also has protective effects on the structure of neurons and vessels in combination therapy of other thrombolytic drugs. The combination of edaravone and argatroban (a selective thrombin inhibitor and an anticoagulant agent used to treat acute noncardioembolic ischemic stroke) protects against damage to neuronal cells and increases the survival ratio in the gerbil stroke model (*p <* 0.05 by Mantel-Cox test) [36]. Edaravone inhibits metalloproteinase 9-related cerebral hemorrhage in MCAO model rats which are treated with recombinant tissue plasminogen activator [51]. Taken together, these findings suggest that edaravone can be used for treating cerebral ischemia by inhibition of the underlying molecular events associated with brain injury, such as an increase in the aquaporin-4 and metalloproteinase 9 expression in astrocytes and in the HMGB1 expression in neuronal cells. In MCAO model rats, which were treated with tissue plasminogen activator, edaravone prevented dissociation of the neurovascular unit (e.g., neurons, glial cells, and vascular cells), dramatically decreased hemorrhagic transformation (hemorrhage that develops inside areas of ischemia), and improved neurological scores and survival rate [52].

## 3. Edaravone Efficacy in Acute Ischemic Stroke

Edaravone has been approved for the treatment of AIS in Japan since 2001; however, it remains under clinical investigation in various other countries [53]. Edaravone is not currently approved for use in Western countries. However, edaravone is recommended by the American Heart Association in the guidelines for the early management of adults with AIS [54]. Clinical trial data show that the administration of edaravone significantly reduces infarct volume when edaravone has been applied within 72 h from the onset of ischemic stroke [55]. The administration of edaravone within 72 h from the onset of ischemic stroke improves neurological outcome over a three-month follow-up period. In Japan, edaravone has been administered within 24 h from the onset of AIS to patients with some stroke subtypes, such as lacunae, large-artery atherosclerosis, and cardioembolic stroke [56–58].

For patients with non-cardioembolic AIS, the Japanese guidelines for the management of stroke in 2009 suggested the use of the antiplatelet agent sodium ozagrel (ozagrel). Ozagrel and edaravone are used in combination in the treatment of patients with non-cardioembolic AIS in Japan. Shinohara *et al.* directly compared the effects of these two medications (edaravone and ozagrel) in 401 patients with AIS in acute non-cardioembolic ischemic stroke (EDO trial) [57]. The intravenous application of edaravone and ozagrel were compared with patient outcome three months after treatment initiation. The rate of patients who were classified as the “no symptoms” or “no significant disability” group was 57.1% and 50.3% for the edavarone medication and ozagrel medication groups, respectively. Therefore, edaravone is at least as effective as ozagrel for the treatment of non-cardioembolic AIS. The results of two clinical trials suggested a correlation between the duration of edaravone therapy and its efficacy. Naritomi *et al.* suggested that the long-term administration of edaravone improves functional outcome of rehabilitation [56] and Unno *et al.* also suggested that long-term administration of edaravone decreases muscle atrophy because of disuse [58].

Edaravone has beneficial effects when used in combination with alteplase. Alteplase, which is a recombinant tissue plasminogen activator, is the only Food and Drug Administration-approved thrombolytic agent for the treatment of AIS [54]. Kimura *et al.* have recently reported that the administration of edaravone during alteplase infusion is likely to enhance recanalization in patients with AIS [59]. Of the 40 patients enrolled in their study, 23 were assigned to the edaravone group (intravenous alteplase infusion with simultaneous intravenous edaravone infusion) and 17 were assigned to the non-edaravone group (intravenous alteplase infusion followed by intravenous edaravone infusion). Early recanalization occurred more frequently in the edaravone group (56.5%) compared with the non-edaravone group (11.8%). In their study, a neurologist determined the National Institutes of Health Stroke Scale (NIHSS) scores before and 24 h after alteplase infusion. Remarkable recovery was defined as a ≥8-point reduction in the total NIHSS score or a total NIHSS score of 0 or 1. Good recovery was defined as a ≥4-point reduction in the total NIHSS score. The rate of patients with “remarkable” or “good” recovery was significantly higher in the edaravone group (80.1%) than in the non-edaravone group (45.5%). Kimura *et al.* hypothesized that the administration of edaravone during alteplase infusion inhibits endothelial cell injury in the occluded artery, and maintains the release of tissue plasminogen activator from endothelial cells, thereby enhancing early recanalization.

## 4. Oxidative Stress and Edaravone Efficacy in Cardiovascular Disease

Oxidative stress is linked with negative outcomes in cardiovascular disease [60]. As discussed above, free radical stress can lead to cardiovascular disease by influencing endothelial function [61]. ROS causes direct cardiac injury by oxidizing cellular constituents and disruption of proteins critical for excitation-contraction coupling, and by diminishing nitric oxide (NO) bioactivity [62].

### 4.1. Atherosclerosis

The majority of cardiovascular disease results from complications of atherosclerosis [63]. Oxidative stress accelerates the progression of atherosclerosis. At sites of plaque growth, NO release is increased in response to the increased shear-stress acting on the vessel wall [64]. This may lead to vasodilation and remodeling of the vessel wall structure [65]. The baseline of vasomotor tone is also decreased in atherosclerotic vessels [66].

Reduction in bioavailability of NO can be inhibited by several mechanisms, including reduction in eNOS expression, lack of substrate or cofactors for eNOS activity, alterations in eNOS cellular signaling, and an increase in NO degradation [67]. Mice with eNOS knockout are more prone to develop typical atherosclerotic lesions in response to adventitial vessel wall injury compared with wild-type mice [68]. Upregulation of tetrahydrobiopterin, which improves bioavailability of NO, has been shown to improve endothelial function and reduce superoxide production [69]. The supplementation of antioxidant, superoxide dismutase, has also been shown to improve endothelium dependent vasodilatation of coronary arteries [70].

In rabbit models of atherosclerosis, edaravone reduces the neointimal thickness and lipid deposition in the subendothelial area, attenuates E-selectin expression and macrophage migration, and proliferates smooth muscle cells [71]. Edaravone significantly inhibits interleukin (IL)-1β-induced proliferation of smooth muscle cells from rabbit aorta, as well as activation of NF-κB. Therefore, edaravone may suppress the development of atherosclerosis. Moreover, edaravone inhibits the nuclear translocation of NF-κB in HUVECs under IL-1β stimulation. Furthermore, edaravone suppresses fatty streak lesions and suppresses macrophages and CD4+ T-cell accumulation and oxidative stress overload in fatty streak lesions in mouse models of atherosclerosis [72]. Similarly, edaravone inhibits H_2_O_2_-induced apoptosis of cultured endothelial cells in parallel with inhibition of 8-isoprostane formation, HNE accumulation, and vascular cell adhesion molecule-1 expression [73]. Edaravone decreases atherosclerotic lesions in the aortic sinus and descending aorta in the mouse models of atherosclerosis. Dihydroethidium labeling and cytochrome c reduction assay have shown that edaravone suppresses the production of superoxide anions in the mouse aorta. Edaravone also reduces plasma 8-isoprostane concentrations and the content of nitrotyrosine, 4-HNE, and vascular cell adhesion molecule-1 in the aorta. Yamaguchi *et al.* reported that edaravone might have clinically beneficial interactions with fluvastatin (lipid-lowering drugs), amlodipine (antihypertensive drugs) and ozagrel regarding the prevention of vascular atherosclerosis on cultured basilar artery smooth muscle cells from guinea-pig [74]. Fluvastatin and amlodipine inhibit the proliferation of the basilar artery smooth muscle cells and co-incubation of edaravone with these drugs augments the antiproliferation effects of these drugs. Ozagrale, GF109203X (a protein kinase C (PKC) inhibitor), Y27632 (a Rho associated coiled-coil forming kinase (ROCK) inhibitor), or edaravone itself does not inhibit the proliferation of the basiral artery smooth muscle cells, but co-incubation of edaravone with ozagrale, GF109203X, or Y37632 induces the antiproliferation effects, which is similler to fluvastatine and amlodipine. Proliferation of vascular smooth muscle cells stimulated by ROS may have pivotal role in the pathogenesis of atherosclerosis. Therefore, PKC and ROCK may contribute to ROS-triggered intracellular signal transduction systems in vascular smooth muscle cells and its proliferation and may also contribute to the pathogenesis of atherosclerosis. This study may stimulate a new research for finding a possible combination therapy of edaravone and various cardiovascular drugs with pleiotropic effects (e.g., fluvastatin and amlodipine).

Scavenging free radicals have a beneficial effect on patients with coronary endothelial dysfunction. In a previous study, 24 patients were divided into two groups on the basis of coronary blood flow responses to acetylcholine. In this study, acetylcholine (30 μg/min) induced an increase in coronary blood flow by more than 300% in patients without coronary risk factors, which indicated a “normal” response group. However, some patients with coronary disease or heart failure did not show the appropriate increase in coronary blood flow by acetylcholine, which indicated an “attenuated” response group [75]. Edaravone improved the acetylcholine-induced increase in coronary blood flow in patients with coronary atherosclerosis. The plasma levels of NO compounds in the attenuated response group were lower than those in the normal response group, and were correlated with the magnitude of coronary blood flow improvement by edaravone. The plasma levels of malondialdehyde and HNE, which indicated the level of oxidative stress, in the attenuated response group were higher than those in the normal response group, and were correlated with the magnitude of coronary blood flow improvement by edaravone. These results suggest that edaravone is a useful therapeutic drug for atherosclerosis, even pending the clinical efficacy.

### 4.2. Diabetes

Diabetes can greatly increase the risk of cardiovascular disease [76]. Many biochemical pathways associated with hyperglycemia increase the production of free radicals [77,78]. Exposure of endothelial cells to a high glucose concentration leads to the augmented production of superoxide radicals [79,80]. Mechanisms of increase in production of superoxide radicals in vascular tissue include uncoupling of eNOS and an increase in nicotinamide adenine dinucleotide phosphate (NADPH) oxidase activity [81,82]. Superoxide radical levels are also increased in hyperinsulinemic rats, which might be related to activation of NADPH oxidase [83]. Measurement of peroxide levels and activities of antioxidant enzymes in the aorta, heart, and blood of streptozotocin-induced diabetic rats showed that oxidative stress starts at an early stage of diabetes and increases progressively [84]. A reduction in antioxidant activity and an increase in oxidative stress occur early after the diagnosis of type I diabetes mellitus in human patients [85]. Hydroxyl radical stress augments an angiotensin II-induced contractile response in diabetic rat thoracic aorta, and edaravone attenuates hydroxyl radical stress and the augmented angiotensin II-induced contractile response in diabetic rats [86]. Edaravone could be an ideal antioxidant adjuvant in the therapy of diabetic vascular complications.

### 4.3. Hypertension

Hypertension can greatly increase the risk of cardiovascular disease [76]. Endothelial dysfunction and an accompanying increase in vascular ROS are observed in most rat models of hypertension [87]. Treatment with antioxidants and superoxide dismutase mimetic drugs attenuate endothelial dysfunction and lower blood pressure in many hypertension models, suggesting that increased ROS production is pathogenic [88]. Furthermore, an increase in oxidative stress by glutathione depletion can cause hypertension. In the hypertensive rat, eNOS and NADPH oxidase were identified as the principle enzymes that produce superoxide radicals [89–91]. Elevation of oxidative stress, which is due to the production of ROS, as well as a decrease in antioxidant systems, has been demonstrated in hypertensive human subjects [92–95]. Hydroxyl radical stress augments angiotensin II-induced contractile responses in the thoracic aorta of spontaneously hypertensive rats. Edaravone could serve as an adjuvant antioxidant therapy for the vascular complications of hypertension by attenuating these enhanced vascular responses [96]. Hypertension induces cardiac hypertrophy and ROS also act as second messengers to develop cardiac hypertrophy in hypertensive model mice [97]. Edaravone significantly attenuates pressure overload–induced cardiac hypertrophy, which is mediated by its antioxidative effect and subsequent inhibition of the apoptosis signal-regulating kinase 1 signaling pathway in the murine model.

### 4.4. Myocardial Infarction

In myocardial ischaemia, hypoxia and reoxygenation induce an increase in free radical production in cardiac tissues and are principal causes of reperfusion injury [64]. ROS, which are produced by the reoxygenation of cardiac tissue, lead to direct oxidative damage to cellular components and also lead to indirect injury via activation of localized inflammation [98]. ROS can also act as signaling messengers in activating biochemical pathways responsible for altering cellular function [99]. For example, exogenous angiotensin II, which leads to the hypertrophy of vascular smooth muscle cells, induces Akt activation in vascular smooth muscle cells. The activation of Akt is mediated by H_2_O_2_, and can be abrogated by overexpression of catalase [100]. Hypoxia/reoxygenation in cardiac myocytes also leads to induction of p38 mitogen-activated protein kinase and JNK pathways, and the activity of these pathways is attenuated by pre-incubation with antioxidants and tyrosine kinase inhibitors [101]. ROS-mediated effects in cardiovascular disease are also reflected in nuclear transcription factor activity. An increase in NF-κB activity has been found in myocardial biopsies of patients with unstable angina [102]. Nuclear translocation of RelA (p65) is also increased in human coronary artery plaques [103].

The pathogenesis of atherosclerosis is thought to be an inflammation-mediated process [104]. Atherosclerosis is associated with increased levels of inflammatory markers, including C-reactive protein, interlekin-6, erythrocyte sedimentation rate, TNF-α, and homocysteine [105]. Hormones and cytokines, such as angiotensin II, platelet-derived growth factor, and TNF-α may increase ROS in atherosclerotic lesions by stimulating local vascular myocytes to produce ROS [106]. Mitochondrial dysfunction and an increase in ROS production are associated with early atherosclerotic lesion formation [107]. Multiple cell populations in the vascular wall have been shown to produce and be regulated by ROS signaling [108]. Free oxygen radicals lead to an increase in oxidization of LDL in the vascular wall and an increase in adhesion molecule expression in endothelial cells, which result in inflammatory cell infiltration and activate matrix metalloproteinases and vascular remodeling [109]. ROS regulate growth and migration of vascular smooth muscle cells in the plaque structure [110]. ROS also trigger extracellular matrix remodeling through regulation of collagen resorption, resulting in compromised plaque stability [109,111].

Reperfusion after myocardial infarction (MI) greatly exacerbates ischemia-related myocardial injury via excessive accumulation of free radicals, which damage the myocardium [112,113]. Edaravone protects against myocardial injury following I/R in patients with AMI [25]. Monocyte chemoattractant protein 1 (also called CCL2) plays an important role in the pathogenesis of acute coronary syndrome [114]. A study demonstrated that edaravone suppresses plasma levels of monocyte chemoattractant protein 1, improves left ventricular ejection fraction, and reduces rehospitalization, which is due to heart failure of patients with AMI. In other studies on AMI, edaravone was reported to reduce infarct size, reperfusion arrhythmia, and levels of serum thioredoxin, a marker of oxidative stress [115], as well as decrease serum concentrations of creatine kinase-MB isoenzymes and improve ventricular ejection [20].

Animal experiments have shown protective effects of edaravone against myocardial I/R injury in an AMI model and in a transplantation model [116–118]. Edaravone reduces the myocardial necrotic area following myocardial I/R in rats [119] and in rabbits [120]. Edaravone also prevents lethal ventricular tachyarrhythmias during reperfusion after MI and deterioration in cardiac function following myocardial ischemia and I/R in rats, by inhibiting lipid peroxidation [121]. In an experimental rat model of coronary occlusion, edaravone reduced the area of MI, maintained adequate myocardial ATP content, decreased mitochondrial swelling, reduced cytochrome-c release, increased the expression of Bcl2, and reduced the number of apoptotic cells and DNA fragmentation [122]. Edaravone also protects cardiac function in rats and reduces infarct size by decreasing the production of TNF-α in the myocardium exposed to I/R injury, and by reducing the release of adhesion molecules, such as P-selectin, from vascular endothelial cells [123]. Recently, Pei *et al.* reported that edaravone may play a role in cardioprotection, partly through modulation of adiponectin levels by suppression of TNF-α [124]. In rabbits, edaravone significantly reduces MI size and improves cardiac function and left ventricular (LV) remodeling by decreasing hydroxyl radicals and superoxide levels in the myocardium, and by increasing the production of NO during reperfusion [113]. Edaravone has also been reported to preserve coronary microvascular endothelial function in dog hearts with I/R injury, which indicates an increase in NO levels, and a decrease in ROS levels [125]. Recently, Miyazaki *et al.* reported in a mouse model that superoxide was produced in myocardium with I/R injury, and the administration of edaravone immediately before reperfusion significantly suppressed superoxide overproduction and subsequent expression of spliced *x-box binding protein-1* and CCAAT/enhancer-binding protein-homologous protein mRNA, followed by reduced injury size. Therefore, the protective effects of edaravone on I/R injury in myocardium are mediated by a reduction in excessive ER stress, and by prevention of direct oxidative damage [126]. Furthermore, the effect of the administration of edaravone on myocardial damage in rabbit hearts subjected to I/R has been examined at different times relative to reperfusion [127]. The administration of edaravone 10 min before reperfusion or immediately upon initiation of reperfusion reduces infarction size and the percentage of apoptotic cells, but treatment with edaravone 5 min after initiation of reperfusion does not show this protective effect. Recently, a novel antioxidant for the potential treatment of ischaemia has been designed by incorporating an isoindoline nitroxide into the framework of edaravone [128]. The administration of the new agent, 5-carboxy-1,1,3,3-tetramethylisoindolin-2-yloxyl, significantly decreases ischemic cell death in a model of ischemia in rat atrial cardiomyocytes in comparison with the administration of edaravone. It is possible that there might be a new antioxidant, which has stronger main effect and smaller side-effect than edaravone.

### 4.5. Heart Failure

The importance of oxidative stress in chronic heart failure can be gauged by the fact that antioxidants prevent the progression of several pathological processes, such as cardiac hypertrophy, cardiac myocyte apoptosis in I/R, and myocardial stunning, which lead to heart failure in animal models [129]. In rat cardiac myocytes, TNF-α and angiotensin II induce hypertrophy in a ROS-dependent manner [130]. Overexpression of catalase significantly reduces angiotensin II-induced hypertrophy, and transfection with antisense p22phox inhibits angiotensin II-induced H_2_O_2_ production [131]. This suggests that NAD(P)H oxidase-induced oxidative stress leads to hypertrophy. During compensated hypertrophy in a guinea pig model, NADPH oxidase-dependent ROS production significantly and progressively increases to the level of decompensated heart failure [132]. This indicates that ROS may be important mediators of heart failure. Other sequelae in the failing heart, including structural damage and contractile dysfunction, may result from xanthine oxidases [133] and failure of mitochondria [134]. Increased production of ROS may decrease NO bioavailability and impair diastolic function [135]. Moreover, increased peroxynitrite may cause cytokine-induced myocardial contractile failure by inactivating sarcoplasmic Ca^2+^-ATPase and dysregulating Ca^2+^ homeostasis [136,137].

Emerging evidence demonstrates that oxidative stress in general, and NADPH oxidase-derived ROS in particular, are important in human cardiac failure [64]. In the failing myocardium of patients with ischemic or dilated cardiomyopathy, NADPH oxidase-derived ROS are upregulated [138]. Plasma TNF-α levels and platelet-derived NADPH oxidase activity are also elevated in patients with heart failure [139]. Plasma ROS levels are often elevated in patients with heart failure [140]. Furthermore, NADPH oxidase activation and increased translocation of regulatory p47phox from the cytosol to the sarcolemmal membrane were recently observed in failing human myocardium [109]. These combined results suggest that oxidative stress has a pathophysiological role in a cardiac dysfunction in heart failure.

During the development of heart failure, edaravone ameliorates defective interdomain interaction of the ryanodine receptor in dogs [141]. Therefore, edaravone prevents Ca^2+^ leakage and LV remodeling, which lead to an improvement in cardiac function and attenuation of LV remodeling. Defective interdomain interaction within the ryanodine receptor appears to play an important role in the pathogenesis of heart failure. Recently, Xin *et al.* reported that edaravone effectively alleviated conduction abnormalities and an increase in serum creatine kinase and aspartate aminotransferase in doxorubicin-induced cardiomyopathy in dogs [142]. Moreover, Arumugam *et al.* reported that edaravone ameliorated the progression of dilated cardiomyopathy by modulating oxidative and ER stress-mediated myocardial apoptosis and fibrosis in rats [143]. These findings indicate that edaravone can reduce or delay the development of heart failure.

### 4.6. Dilated Cardiomyopathy

Autoimmune responses and inflammation are involved in the pathogenesis of many cardiovascular diseases. Dilated cardiomyopathy is a leading cause of heart failure and frequently results from postinfectious autoimmunity [144]. Similar phenomena are frequently observed in myocarditis and dilated cardiomyopathy, such as myocardial infiltration of lymphocytes and mononuclear cells, an increase in expression of pro-inflammatory chemokines and cytokines, and circulating autoantibodies. Experimental autoimmune myocarditis (EAM) in rodents can be elicited by immunization of cardiac myosin. EAM in rats mimics human fulminant myocarditis in their acute phase and mimics human dilated cardiomyopathy in their chronic phase.

In rats with acute EAM, edaravone reduces the number of IL-1β-positive cells [145]. In this animal model, edaravone reduces myocardial IL-1β-positive cells and myocardial oxidative stress overloads with DNA damage. Edaravone also decreases the carbonyl content of myocardial protein, myocardial thiobarbituric acid reactive substances, the formation of hydroxyl radicals, and the cytotoxic activity of lymphocytes. Thiobarbituric acid reactive substances are formed as a byproduct of lipid peroxidation [146]. Because ROS have extremely short half-lives, they are difficult to measure directly. Therefore, thiobarbituric acid reactive substances are measured as a marker for oxidative stress. Furthermore, edaravone protects against acute EAM by scavenging hydroxyl free radicals and reducing oxidative stress, which ultimately suppress autoimmune-mediated myocardial damage [147]. In another study, edaravone was reported to ameliorate the progression of EAM, to improve LV function, to decrease nicotinamide adenine dinucleotide phosphate oxidase subunit p67-phox expression and ER stress signaling proteins (GRP78 and caspase 12) in the LV, and to reduce the number of terminal deoxynucleotidyl transferase-mediated dUTP nick end labeling-positive cells in the rat cardiac ventricle with EAM [148]. Recently, Arumugam *et al.* reported that mitogen-activated protein kinase signaling, as well as AMP-activated protein kinase signaling, play a crucial role in the progression of EAM, and this can be effectively blocked by treatment with edaravone in rats [149]. These findings suggest that ROS induce autoimmune-mediated damage, which is probably mediated by the production of cytokines. Therefore, edaravone could prevent the development of dilated cardiomyopathy.

### 4.7. Other Cardiovascular Disease

Cardioplegic arrest is the main technique used for myocardial protection during open-heart surgery; however, it can lead to myocardial injury during reperfusion [118]. Free radical scavengers attenuate I/R injury in various settings, and the addition of edaravone to cardioplegic solution attenuates myocardial dysfunction following cardioplegic arrest in rats by the suppression of oxidative stress. Edaravone also exerts cardioprotective effects in a pig heart transplantation model by the inhibition of lipid peroxidation [117]. *In vitro*, edaravone reduces I/R-induced cell death by the attenuation of ROS production in rabbit cardiomyocytes [150]. Therefore, edaravone could protect cardiomyocytes against myocardial injury during reperfusion after cardioplegic arrest.

## 5. Side Effects of Edaravone

Side effects, such as acute renal failure, liver dysfunction, acute allergic reactions, disseminated intravascular coagulation, thrombocytopenia, and leukocytopenia, are occasionally observed by >5% of patients during edaravone treatment [25]. Therefore, edaravone can be used with a low rate of side effects. No side effects are anticipated unless edaravone is used in elderly patients with renal dysfunction.

## 6. Conclusions

In neurological disease, edaravone principally acts as a free radical scavenger to protect against I/R-induced injury. In this review, we suggest that edaravone has beneficial effects on myocardial and vascular injury following I/R in patients with AMI, as well as vascular injury in atherosclerosis, diabetes, and hypertension in the chronic phase, and myocardial injury in heart failure and dilated cardiomyopathy. The results of the studies discussed in this review point towards multiple mechanisms of action of edaravone, which are attributable, at least in part, to its anti-oxidant activity, similar to that in neural injury, in addition to several pleiotropic effects. For example, edaravone suppresses the increase in circulating free radical levels and markers of ROS generation associated with I/R injury. Furthermore, edaravone targets numerous intracellular signaling pathways suppressing the release of pro-inflammatory cytokines and activation/infiltration of inflammatory cells, such as macrophages. Prospective studies using a large population of patients are required to evaluate the effects of edaravone in clinical settings, and determine whether edaravone improves the prognosis of cardiovascular diseases. Moreover, the timing of edaravone administration (before, during or after reperfusion treatment) is extremely important. Edaravone would be useful for treatment of various cardiovascular diseases in which oxidative stress may be involved in the pathogenesis.

## Abbreviations

AIS: Acute ischemic stroke
AMI: acute myocardial infarction
EAM: experimental autoimmune myocarditis
EDO trial: edaravone *vs.* sodium ozagrel in acute non-cardioembolic ischemic stroke
eNOS: endothelial nitric oxide synthase
ER: endoplasmic reticulum
HMGB1: high mobility group box 1
HNE: 4-hydroxy-2-nonenal
HUVEC: human umbilical vein endothelial cell
IL: interleukin
iNOS: inducible nitric oxide synthase
I/R: ischemia and reperfusion
LDL: low-density lipoprotein
LV: left ventricle
MAP: mitogen activated protein kinase
MCAO: middle cerebral artery occlusion
MI: myocardial infarction
NADPH: nicotinamide adenine dinucleotide phosphate
NIHSS: National Institutes of Health Stroke Scale
NF-κB: nuclear factor-κB
NO: nitric oxide
PKC: protein kinase C
ROCK: Rho associated coiled-coil forming kinase
ROS: Reactive oxygen species
TNF-α: tumor necrosis factor α
WHO: World Health Organization.
